# Awareness on passive smoking among Indian adults

**DOI:** 10.6026/97320630019010

**Published:** 2023-01-31

**Authors:** Sivasubramanian N, Mahalakshmi B, Patel Shivani Arvindbhai, Ramalakshmi G

**Affiliations:** 1 Nootan College of Nursing, Sankalchand Patel University, Visnagar, Gujarat - 384315, India; 2 College of Nursing, SGRR University, Dehradun, Uttarkhand - 248001, India

**Keywords:** Awareness, Adverse Effects, Passive Smoking, Adults

## Abstract

Around one-third of adults worldwide regularly come into contact with passive smoking. In addition to many other health issues, non-smokers who are exposed to passive smoking at home or at work have a 20–30% higher risk of acquiring lung cancer. The current study set out to determine adult smokers' awareness of the hazards of passive smoking as well as the efficacy of an education campaign in raising that awareness. To finish the investigation, a pre-experimental research approach was employed. 60 adults between the ages of 18 and 45 were chosen by non-probability purposive sampling. Results revealed that the mean of pre-test score was 12.33 and mean of post-test score was 21.5. The mean difference was 9.17. The standard deviation of pre-test knowledge score was 2.87 and standard deviation post-test knowledge score was 3.27. The calculated 't' value is 14.80, the DF value was 59 and p value was 1.671. This shows that the teaching program was effective in increasing the awareness of adults regarding effects of passive smoking. Age and education status of the sample were found to have significant association with the level of awareness in pre-test. Teaching programs can be effectively utilized to create awareness among adults regarding adverse effects of passive smoking.

## Background:

Worldwide, smoking poses a serious threat to public health. The effects of passive smoking on health have not been thoroughly analysed and are still mostly uncertain. Existing research indicates that smoking, whether active or passive, may raise the risk of developing a number of illnesses, including food allergies, allergic rhinitis, allergic dermatitis, and female breast cancer [1]. According to the findings of a meta-analysis review study, passive smoking raises the risk of colorectal cancer [2]. In a publication, primary studies and meta-analyses from residential and office settings provided a wealth of evidence showing non-smokers exposed to passive smoking have a higher chance of developing lung cancer [3]. Smoking has been linked to an increased risk of allergic illnesses in both children and adults, with passive smoking being linked to an increased risk of food allergies [4]. According to a meta-analysis of 4 prospective cohort studies, passive smoking is significantly linked to an elevated risk of type 2 diabetes [5]. The side stream smoke released from the cigarette's burning tip is what causes the majority of passive smoking exposure. Due to the high levels of ammonia, benzene, nicotine, carbon monoxide, and other carcinogens, side stream smoke is dangerous. Chronic passive smoking exposure is thought to provide health hazards to non-smokers that are comparable to those of a light smoker. Adults who already have previous medical issues including allergies, chronic lung diseases, or angina have their symptoms made worse by exposure to environmental tobacco smoke. In healthy adults, headaches, nausea, and irritation of the eyes and nasal mucous membranes are among the acute health effects [6]. Every year, 600,000 people are killed by passive smoking or second hand smoke (SHS). Around one-third of adults worldwide are regularly exposed to SHS. In India, 52% of adults were exposed to SHS at home (rural 58%, urban 39%) [7]. According to a study on the health consequences of second hand smoke from cigarettes among adult primary care patients who have never smoked, the existing level of awareness should be enhanced while efforts should be stepped up to address areas where the level of awareness is low[8].Creating public health awareness and educating all healthcare professionals about tobacco control and cessation by including the topic in curriculum is crucial in controlling the negative effects of active and passive smoking, according to a review paper that thoroughly covered all of the various aspects of tobacco control in the Indian context[7].The results of a cross-sectional Internet survey with 1,128 men and 458 women aged 15 to 71 years old to examine the relationship between smokers' awareness of the harm caused by second hand smoke and their interest in quitting smoking suggest that increasing smokers' awareness of the harm caused by second hand smoke may increase their interest in quitting. The data can be used to promote tobacco control in the future [9].The research studies on the awareness of the adverse effects of smoking has been conducted worldwide in sufficient numbers, but the studies emphasizing the hazards of passive smoking are less and rare, especially among Indian Adults. Hence it is of interest to assess the awareness regarding effects of passive smoking and to improve the awareness through an education program.

## Methodology:

The current study set out to determine adults' awareness of the hazards of passive smoking as well as the efficacy of an education campaign in raising that awareness. To finish the investigation, a pre-experimental research approach was employed. The study comprised a sample of 60 persons who fit the requirements, such as being between the ages of 18 and 45. The sample was chosen using the non-probability purposive sampling technique. The study was carried out in the following order;

E1 - Pre-test level of awareness regarding effects of passive smoking

X -Education program on effects of passive smoking

E2 - Post-test level of awareness regarding effects of passive smoking

 A structured questionnaire designed to measure the awareness of adults regarding effects of passive smoking was used to collect data from the sample. In this study, the term education program refers to a health teaching to the selected sample regarding effects of passive smoking. This teaching contains:

[1] Meaning of active and passive smoking

 [2] Contents of smoke from a cigarette

[3] Body systems affected by smoking

[4] Adverse effect of passive smoking on various body systems

[5] Chronic diseases caused by passive smoking

[6] Methods to stop smoking habit

[7] Ways to prevent passive smoking

Prior to education program, pre-test was conducted using structured questionnaire. Then the health teaching was provided to all samples using Charts, PPT and Pamphlets. After one week of the education program the post intervention level of awareness was measured using same structured questionnaire to evaluate the effectiveness of education program. Several statistical techniques were used to examine and interpret the data that was gathered.

## Results:

Majority of sample (43.3%) were belongs to the age group 36-45 years. Sample contains 50% male and 50% female. Out of 60 samples 40 had only primary education and only 5 were graduates. 33% of study samples were living in joint family. Parents of 36.67 % have habit of smoking and 42.3% samples were agreed that they used to smoke.

**Figure 1:**
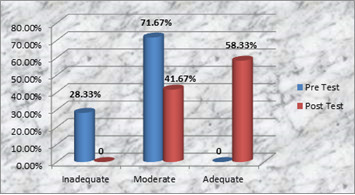
Cylinder diagram depicting percentage distribution of the samples according to the pre-test and post-test level of knowledge

Figure 1 shows that prior to the administration of teaching programme, in the pre-test (28.33%) of the all sample had poor knowledge, 71.67% had moderate knowledge and no one had adequate knowledge. In the post test there was marked improvement in the knowledge of the sample with (41.67%) gained moderate knowledge and (58.33%) gained good knowledge. The data presented in Table 1 states that the mean of pre-test score was 12.33 and mean of post-test score was 21.5. The mean difference was 9.17. The standard deviation of pre-test knowledge score was 2.87 and standard deviation post-test knowledge score was 3.27. The calculated 't' value is 14.80, the DF value was 59 and p value was 1.671. The calculated 't' value (14.80) was greater than the table value (1.67) at 0.05 level of significance that shows the teaching program was effective in increasing the awareness of adults regarding effects of passive smoking. Age and education status of the sample were found to have significant association with the level of awareness in pre-test.

## Discussion:

The current study set out to determine adult smokers' awareness of the hazards of passive smoking as well as the efficacy of an education campaign in raising that awareness. The study's findings showed that adults' knowledge of the negative effects of passive smoking was either average or insufficient. No sample had scored well enough to indicate acceptable awareness. A cross-sectional study conducted in February 2020 in Saudi Arabia to examine medical students' level of knowledge about the dangers of second hand smoke found that their level of understanding is insufficient [10]. 400 undergraduate students were randomly chosen for a cross-sectional study to determine the prevalence of passive smoking exposure and knowledge of its negative effects. According to their study's findings, adolescents were more aware than adults (86.5%) of the negative effects of passive smoking [11]. The findings of the current study showed that the education campaign was successful in raising public knowledge of the harmful consequences of passive smoking. In a few colleges in Rajkot, a study was done to determine the effectiveness of a structured teaching programme on the risks of smoking in terms of knowledge among adolescent boys. This study also demonstrated that a teaching programme was successful in raising awareness of the harmful effects of smoking [12]. Another experimental study, which assessed the efficiency of a programme for teaching adults about tobacco abuse, showed that the programme was successful in raising adults' understanding of the subject [13]. The present study also found that there was significant association between age and educational status of samples with their level of awareness. Another study on the harmful consequences of active and passive smoking in teenage boys demonstrates that certain demographic factors, such as age, education, and prior knowledge of the hazards of smoking, show a strong relationship with their level of knowledge [14].

## Conclusion:

In addition to numerous other health issues, non-smokers who are exposed to passive smoking at home or at work have a 20-30% higher risk of acquiring lung cancer [15]. The awareness of adults regarding effects of passive smoking was average or inadequate. The findings of this study showed that the educational programme was successful in raising awareness of the harmful consequences of passive smoking. Further studies need to be conducted to identify the ill effects of passive smoking among non-smokers living with smokers. Educational curriculum for health professionals should be included with training for awareness programs on effects of active and passive smoking.

## Figures and Tables

**Table 1:** Mean, Standard Deviation and 't' value of pre-test and post-test knowledge scores of effectiveness of teaching program. DF= n-1 (60-1) =59

Parameter	Mean	S.D	Mean Difference	t-value	Result
Pre-test	12.33	2.87	9.17	14.80*	Sig. <0.05
Post-test	21.5	3.27			
